# Changes of Regional Neural Activity Homogeneity in Preclinical Alzheimer’s Disease: Compensation and Dysfunction

**DOI:** 10.3389/fnins.2021.646414

**Published:** 2021-06-17

**Authors:** Zhen Zhang, Liang Cui, Yanlu Huang, Yu Chen, Yuehua Li, Qihao Guo

**Affiliations:** ^1^Department of Gerontology, Shanghai Jiao Tong University Affiliated Sixth People’s Hospital, Shanghai, China; ^2^The Brain Cognition and Brain Disease Institute, Shenzhen Institute of Advanced Technology, Chinese Academy of Sciences, Shenzhen–Hong Kong Institute of Brain Science-Shenzhen Fundamental Research Institutions, Shenzhen, China; ^3^Department of Radiology, Shanghai Jiao Tong University Affiliated Sixth People’s Hospital, Shanghai, China

**Keywords:** subjective cognitive decline, mild cognitive impairment, regional homogeneity, resting-state functional MRI, cognitive function

## Abstract

**Introduction:**

Subjective cognitive decline (SCD) is the preclinical stage of Alzheimer’s disease and may develop into amnestic mild cognitive impairment (aMCI). Finding suitable biomarkers is the key to accurately identifying SCD. Previous resting-state functional magnetic resonance imaging (rs-fMRI) studies on SCD patients showed functional connectivity disorders. Our goal was to explore whether local neurological homogeneity changes in SCD patients, the relationship between these changes and cognitive function, and similarities of neurological homogeneity changes between SCD and aMCI patients.

**Materials and Methods:**

37 cases of the healthy control (HC) group, 39 cases of the SCD group, and 28 cases of the aMCI group were included. Participants underwent rs-fMRI examination and a set of neuropsychological test batteries. Regional homogeneity (ReHo) was calculated and compared between groups. ReHo values were extracted from meaningful regions in the SCD group, and the correlation between ReHo values with the performance of neuropsychological tests was analyzed.

**Results:**

Our results showed significant changes in the ReHo among groups. In the SCD group compared with the HC group, part of the parietal lobe, frontal lobe, and occipital lobe showed decreased ReHo, and the temporal lobe, part of the parietal lobe and the frontal lobe showed increased ReHo. The increased area of ReHo was negatively correlated with the decreased area, and was related to decrease on multiple neuropsychological tests performance. Simultaneously, the changed areas of ReHo in SCD patients are similar to aMCI patients, while aMCI group’s neuropsychological test performance was significantly lower than that of the SCD group.

**Conclusion:**

There are significant changes in local neurological homogeneity in SCD patients, and related to the decline of cognitive function. The increase of neurological homogeneity in the temporal lobe and adjacent area is negatively correlated with cognitive function, reflecting compensation for local neural damage. These changes in local neurological homogeneity in SCD patients are similar to aMCI patients, suggesting similar neuropathy in these two stages. However, the aMCI group’s cognitive function was significantly worse than that of the SCD group, suggesting that this compensation is limited. In summary, regional neural activity homogeneity may be a potential biomarker for identifying SCD and measuring the disease severity.

## Introduction

Alzheimer’s disease (AD) is the most common cause of dementia in the elderly. The pathophysiological changes leading to AD have begun years or even decades before AD symptoms appear (Sperling et al., 2011). According to the recommendations from US National Institute on Aging-Alzheimer’s Association (NIA-AA), the progress from normal to AD can be divided into three stages: (1) the preclinical stage, (2) the mild cognitive impairment (MCI) stage, and (3) dementia stage (Jack et al., 2011). In these stages, neuronal damage and cognitive decline progress continuously and irreversibly. Therefore, it is essential to identify potential patients with AD as early as possible.

Subjective cognitive decline (SCD) is considered the last stage of the preclinical stage of AD. SCD refers to individuals subjectively perceive the decline of their memory or other cognitive abilities compared with their previous cognitive ability (Jessen et al., 2014). The decline is gradually developed and not caused by any acute events, while there is no objective cognitive impairment. Performing neuropsychological testing on people with SCD will find that they do not meet the MCI diagnostic criteria. At this stage, individuals suffer only mild neuropathological damage and still have a considerable cognitive reserve; therefore, SCD is considered a critical window for intervention to prevent individuals from progressing to AD (Jessen et al., 2014; Rabin et al., 2017).

If the neuropathological changes continue to deteriorate, individuals with SCD may progress to MCI. MCI is an early but objective state of cognitive impairment (Petersen, 2004; Albert et al., 2011), in which the amnestic mild cognitive impairment (aMCI) subtype is closely related to AD. The aMCI patients’ general cognitive function is impaired, with memory function as the primary manifestation. Although still retain roughly intact functional activities, patients with aMCI have a high conversion rate to AD (Mitchell and Shiri-Feshki, 2009).

To accurately identify potential AD patients, it is necessary to select appropriate biomarkers, which is particularly important for SCD patients because they have no obvious abnormal neuropsychological test performance at this stage. At present, the core biomarkers in AD are mainly divided into cerebrospinal fluid and imaging biomarkers (Scheltens et al., 2016). cerebrospinal fluid biomarkers mainly including Aββ42, total tau, and phosphorylated tau, while imaging biomarkers mainly including Aβ42 and tau PET CT. The biomarkers in cerebrospinal fluid have good sensitivity (Shaw et al., 2009; Visser et al., 2009) but can only be detected by invasive examination, making it difficult for these biomarkers to be widely used. Therefore, we need more non-invasive markers. Resting-state functional magnetic resonance imaging (rs-fMRI) is a method to explore the functional activity of the brain; much progress has been made in the use of rs-fMRI in the fields of MCI and AD, proving that there are significant brain function changes in these stages (Pan et al., 2017; Bi et al., 2020a,b; Moguilner et al., 2020). In the field of SCD, rs-fMRI has also been used, mainly focused on brain network connectivity changes. A previous study (Dillen et al., 2017) has shown that the functional connectivity among nodes in the default mode network of SCD patients is weakened; the connectivity between the default mode network and hippocampus is also affected. These changes in connectivity are related to the decline of memory ability. Another study (Viviano et al., 2019) found that the posterior memory network’s connectivity in patients with SCD also decreased, but no significant changes were found in simultaneous diffusion-weighted image analysis. A study of SCD using machine learning (Yan et al., 2019) confirmed changes in the default network connectivity and found changes in the subcortical structure network. Based on these studies on large-scale network connectivity, a recent study (Wang et al., 2019) went one step further and found primary medium-scale network damage in SCD patients. Some studies found the correlation between rs-fMRI and classical pathological biomarkers in the preclinical stage of AD, which suggest that fMRI can be considered a potential imaging biomarker. A study using amyloid-PET, FDG-PET, and fMRI found left frontal cortex connectivity underlies cognitive reserve in prodromal Alzheimer disease, suggested that functional changes in the prodromal stage of AD are consistent with the pathological changes (Franzmeier et al., 2017). Another study on MCI found a correlation between local functional activity and the Aβ/p Tau ratio of cerebrospinal fluid, which may be a sensitive indicator of AD pathology (Ren et al., 2016). Using machine learning to analyze the fMRI and cerebrospinal fluid biomarkers of SCD individuals in the ADNI database, researchers found that SCD individuals showed higher nodal topological properties associated with Aβ levels and memory function, suggested the compensatory mechanism of the functional connectivity (Chen et al., 2020). A subsequent RS-fMRI study based on the DELCODE cohort suggested that local brain function changes in patients with SCD were associated with Aβ load (Li et al., 2021).

Previous studies have revealed changes in the strength of connectivity in SCD patients but did not explain why these changes occur. To explore the possible mechanisms behind connectivity changes, we first need to find a suitable local brain function indicator. Regional homogeneity (ReHo) (Zang et al., 2004) is a stable indicator to detect regional synchronization and can be used to evaluate the regional neural activity homogeneity. Using Kendall’s coefficient concordance, ReHo can evaluate the time series similarity between local voxels and adjacent voxels. The abnormality of ReHo, including decrease or increase, may reflect the disorder and compensation of local brain function and may explain the internal cause of whole-brain network disorder (Zuo et al., 2013; Xiong et al., 2020). With these characteristics, ReHo is a very suitable indicator to study the local brain function changes in patients with SCD. In addition, A regional functional synchronization study found that ReHo might have distinctive association patterns with Aβ retention in elders with normal cognitive (Kang et al., 2017). By measuring ReHo and biomarkers, researchers found the ReHo in different regions of aMCI patients is related to cognitive function and cerebrospinal fluid Aβ42 level (Luo et al., 2018). These studies suggested that in the preclinical stage of AD, regional neural activity homogeneity may be related to pathological changes to some extent.

The aim of this study was to reveal the regional neural activity homogeneity changes in patients with SCD and the significance of these changes. We used rs-fMRI ReHo to compare the differences between SCD patients and normal subjects. In order to verify whether SCD is a preclinical stage of AD, we also included patients with aMCI for the same analysis to find out whether there is a similarity between the SCD and aMCI groups. In order to clarify the relationship between regional neural activity homogeneity and cognitive function, we used a variety of neuropsychological tests to analyze the correlation with ReHo.

Based on the current existing facts: (1) SCD is a preclinical state of neurodegenerative disease; there are local neuropathological changes at this stage, and (2) the neuropsychological manifestations of SCD patients are still roughly within the normal range. We make the following hypothesis: (1) there are corresponding changes in regional brain function in patients with SCD, and the scope of this change is large enough to affect large-scale brain functional connectivity; (2) generally normal cognitive function in SCD patients may be due to a certain degree of functional compensation; (3) as the precursor stage of aMCI, the changes of regional brain function in SCD may be similar to aMCI to some extent; (4) in patients with SCD, there may be a correlation between their cognitive ability and these regional brain function changes, which will lead to a gradual decline in their cognitive function if the changes continue to progress.

## Materials and Methods

### Participants

Participants were recruited from the community through advertising between August 2018 to November 2019. The recruitment was carried out in the neuropsychological testing room of the Department of Geriatrics, Shanghai Jiao Tong University Affiliated Sixth People’s Hospital, Shanghai, China. A total of 104 participants were included.

#### The Healthy Control Group (HC Group)

HCs were additionally required to have no significant impairment in cognitive function, no memory complaints or memory loss observed, MMSE score ≥ the cutoff (Katzman et al., 1988), a CDR score of 0 (Morris, 1993), and a Hamilton Depression Rating Scale score of 12 or less in the past 2 weeks (Worboys, 2013). MRI manifestations: no key parts such as thalamus and hippocampal infarction; no white matter damage (Fazekas Scale ≥ 3) (Fazekas et al., 1987).

Thirty-seven healthy participants were classified as the HC group [15 men; age: mean = 63.86 years, standard deviation (SD) = 8.25 years; the number of years of full-time education: mean = 12.11 years, SD = 3.42 years; Mini-Mental State Examination (MMSE): mean = 28.57, SD = 1.21].

#### The Subjective Cognitive Decline Group (SCD Group)

The diagnosis criteria of SCD was based on features referred to SCD plus (preclinical AD) (Jessen et al., 2014): (a) subjective decline in memory, rather than other domains of cognition; (b) onset of SCD with the last five years; (c) concerns (worries) associated with SCD; (d) feeling of worse performance than others of the same age group; (e) normal performance on Neuropsychological scale and did not reach the criteria for MCI or dementia. We used the SCD-initiative (SCD-I) framework to include individuals with SCD (Jessen et al., 2018; Miebach et al., 2019), who have the following performance: Reported subjective cognitive decline (worse than peers) and worried about it; the first occurrence of subjective cognitive decline was less than 5 years before the interview; after adjusting for age, sex and education, compared with HCs, the score difference of each test in the neuropsychological battery was less than 1.5 standard deviations.

Thirty-nine patients diagnosed with SCD were included (14 men; age: mean = 64.56 years, SD = 7.34 years; number of years of full-time education: mean = 11.69 years, SD = 3.30 years; MMSE: mean = 27.90, SD = 1.94).

#### The Amnestic Mild Cognitive Impairment (aMCI Group)

The inclusion criteria for aMCI was referred from the criteria proposed by Jak/Bondi (Bondi et al., 2014): (1) Cognitive concern or complaints by the subject, informant, nurse, or physician during the last year; (2) Mini-Mental State Examination (MMSE) above cut-off (> 24/30); (3) objective memory impairment assessment by long-delay free recall and recognition of Auditory Verbal Learning Test (AVLT) in at least 1.0 standard deviation (SD) below the norm for age and education; (4) Maintained activities of daily living or slight impairment in instrumental activities of daily living, in other words, no more than one item from the Activities of Daily Living Scale (ADL)-Chinese version suffered obvious changes; (5) Absence of dementia, according to the NIA-AA criteria.

Twenty-eight patients diagnosed with aMCI were included (13 men; age: mean = 65.71 years, SD = 6.90 years; number of years of full-time education: mean = 12.19 years, SD = 3.16 years; MMSE: mean = 27.07, SD = 1.72).

#### The Exclusion Criteria

The exclusion criteria were: (a) patients diagnosed or with a history of head injury, head surgery, mental diseases, brain tumors, acute cerebral hemorrhage, cerebral ischemia, non-degenerative brain injury; (b) patients with severe visual or hearing impairment; and (c) patients who could not undergo MRI. To exclude other possible causes for the amnestic impairment, each subject had a uniform structured evaluation performed by a neurologist, which included a medical history inquiry and neurological examination. Blood tests included complete blood count, thyroid function tests, serum vitamin B12, and Venereal Disease Research Laboratories test.

### Neuropsychological Assessments

All participants underwent extensive neuropsychological tests, included: MMSE (total score: 30) (Folstein et al., 1975), Addenbrooke’s Cognitive Examination (ACE-III) (total score: 100) (Mioshi et al., 2006), Montreal Cognitive Assessment-Basic (MoCA-B) (total score: 30) (Huang et al., 2018b), Auditory Verbal Learning Test (AVLT) (score: 12 per round, immediate recall score equals the sum of the first, second and third recall scores, recognition score: 24) (Zhao et al., 2015), Brief Visuospatial Memory Test (BVMT) (score: 12 per round, immediate recall score equals the sum of the first, second and third recall scores) (Pliskin et al., 2020), Animal Verbal Fluency Test (AFT) (Zhao et al., 2013a), Boston Naming Test (BNT) (total score: 30) (Mack et al., 1992), Silhouettes Test (ST) (total score: 15) (Huang et al., 2018a), Shape Trail Test (STT) (Zhao et al., 2013b), Stroop Test (total score: 24) (Chen et al., 2019), Judgment of Line Orientation (JLO) (total score: 30) (Qualls et al., 2000), and Digit Span Test (DST) (sequence score: 12; reverse score: 10) (Johansson and Berg, 1989).

### Functional Magnetic Resonance Imaging

#### Image Acquisition

Resting-state fMRI was performed with a 3.0-Tesla scanner (SIEMENS MAGNETOM Prisma 3.0T, Siemens, Erlangen, Germany). parameters were: echo-planar imaging (EPI) sequence, transverse plane, repetition time = 800 ms, echo time = 37 ms, flip angle = 52 °, matrix size = 104 × 104, field of view = 208 mm × 208 mm, slice number = 72 slices, slice thickness = 2 mm, and voxel size = 2 mm × 2 mm × 2 mm. The scan obtained 488 slices and took a total of 404 s. During the entirety of the scan, the participants were asked to lie in the scanner, close their eyes but not fall asleep, try to keep their heads still, and not to think systematically.

#### Imaging Data Processing

The data were processed using Statistical Parametric Mapping 12 (SPM12)^1^ and RESTplus^2^ toolkits (Jia et al., 2019). In order to stabilize the magnetic field of the MRI scanner and allow the participant to adapt to the noise, the first 20 time points were removed. Next, the following preprocessing steps were carried out: slice timing to correct differences in image acquisition time between slices, realignment for the correction of head motion (excessive head movement: ≥ 3 mm or 3°), spatially normalized to the Montreal Neurological Institute (MNI) space and resampled to 3 mm isotropic voxels, remove linear and quadratic trends of the time-series signals, regress out the white matter, cerebrospinal fluid, global mean signal, and Friston-24 motion parameters, and band-pass (0.01–0.08 Hz) filter.

The ReHo was obtained by calculating the Kendall coordination coefficient of the time process for each of the 27 nearest neighboring voxels and then standardized by dividing each voxel’s value by the global average. Finally, the standardized mean ReHo graphs were spatially smoothed using a Gaussian kernel (FWHM = 6 mm).

### Statistical Analysis

SPSS (IBM SPSS Statistics, Version 26.0. IBM Corp, Armonk, NY, United States) software was used to analyze demographic data and neuropsychological test scores. Data were tested for normality using a Shapiro-Wilk normality test. Normally distributed data were presented as means ± SD. The non-normally distributed data were expressed as the median (quartile range). Pearson Chi-Square test was used to test the differences for sex, hypertension, hypercholesterolemia, and diabetes. The analysis of variance (ANOVA) was used to analyze the age, education years, and neuropsychological test scores conformed to normality among the three groups. For neuropsychological test scores that do not conform to the normal distribution, a non-parametric test (Kruskal-Wallis H-test) was performed among the three groups. In *post-hoc* analysis, Bonferroni’s correction was applied when multiple comparisons were performed. To analyze the correlation between neuropsychological tests and ReHo values, we conducted Spearman rank correlation analysis.

SPM12 was used to establish a statistical model to analyze the differences in ReHo. ANOVA analysis was carried out among the three groups to determine the areas where there were differences. The independent-sample *t*-test was then carried out for comparing ReHo between the SCD group with the HC group and the aMCI group with the HC group. False Discovery Rate (FDR) correction for multiple comparisons was performed (*p* < 0.001, *k* > 10 voxels) using RESTplus toolkits. To separately show the differences between the SCD group compared with the HC group and the aMCI group compared with the HC group, we performed whole-brain two-sample *t*-tests. In order to display the results of two *t*-tests together without increasing the false-positive rate, the FDR correction threshold was adjusted to 0.0005.

Compared with the HC group, the significant clusters that survived after the multiple comparison correction were defined as regions of interest (ROIs). If only one cluster survived, it was considered the significant cluster and chosen as ROI. If multiple clusters survived, the cluster with the highest peak *t*-value and the largest volume was considered the most significant and was defined as the ROI. The ReHo values of ROIs were extracted and used for Spearman rank correlation analysis.

The imaging results were visualized using BrainNet Viewer^3^ (Xia et al., 2013) and RESTplus.

## Results

### Demographic Data and Neuropsychological Performances

#### Comparison of Demographic Data Between Groups

There were no statistical differences in age, sex, and education years among the three groups. There were no statistical differences in hypertension (24.3% in HC group, 28.2% in SCD group, 32.1% in aMCI group), hypercholesterolemia (8.1% in HC group, 12.8% in SCD group, 10.7% in aMCI group), and diabetes (13.5% in HC group, 10.2% in SCD group, 14.3% in aMCI group) among the three groups.

#### Comparison of Neuropsychological Performance Between Groups

Multi-group comparisons found differences in MMSE, ACE-III, MoCA-B, AVLT immediate recall, AVLT 4th recall, AVLT 5th recall, AVLT 6th recall, AVLT recognition, BVMT immediate recall, BVMT 6th recall, BVMT recognition, AFT, BNT, STT-A total time, and DST sequence (Table 1). There were no significant difference between the three groups in BVMT-4th recall, BVMT-5th recall, ST, STT-B total time, JLO (Table 1). *Post-hoc* analysis with Bonferroni’s correction was conducted to confirm differences occurred between groups.

**TABLE 1:** Demographic data and neuropsychological tests between groups.

	HC (*n* = 37)	SCD (*n* = 39)	aMCI (*n* = 28)	Test statistic
Age (year)^a^	63.86 ± 8.250	64.56 ± 7.337	65.71 ± 6.895	0.478
Sex^b^	Male = 15	Male = 14	Male = 13	0.751
Edu years (year)^a^	12.108 ± 3.422	11.692 ± 3.300	12.185 ± 3.1627	0.227
Hypertension^b^	24.3%	28.2%	32.1%	0.488
Hyper- cholesterolemia^b^	8.1%	12.8%	10.7%	0.447
Diabetes^b^	13.5%	10.2%	14.3%	0.296
**General cognitive function**				
MMSE^c^	29 (28,30)	28 (27,29)	27 (26,28)*	12.069
ACE-III^c^	87 (82.5,90.5)	82 (77,86)*	75 (73,81)**^†^	26.792
MoCA-B^c^	27 (26,28)	26 (23,28)	23 (22,25)**^†^	25.177
**Memory function**				
AVLT immediate recall^c^	18 (15.5,20.5)	19 (15,21)	12 (11,14)**^‡^	37.136
AVLT 4th recall^c^	6 (5,8)	6 (5,8)	3 (2,2.75)**^‡^	47.123
AVLT 5th recall^c^	6 (4.5,7.5)	6 (5,7)	2 (1,3)**^‡^	49.797
AVLT 6th recall^c^	6 (5,7.5)	5 (4,7)	2 (2,3)**^‡^	44.665
AVLT recognition^c^	22 (21,23)	22 (21,24)	18 (16,18.75)**^‡^	55.987
BVMT immediate recall^c^	22 (16.5,26)	21 (17,25)	18 (14.25,20.75)*	6.586
BVMT 4th recall^c^	10 (7.5,11.5)	10 (8,11)	8 (5.25,10)	6.074
BVMT 5th recall^c^	10 (8,11.5)	10 (8,11)	8 (5.25,10)	6.269
BVMT 6th recall^c^	5 (5,6)	5 (5,6)	4 (4,4)**^‡^	23.940
BVMT recognition^c^	12 (12,12)	12 (12,12)	12 (10,12)**^†^	20.005
**Language function**				
AFT^c^	19 (18.5,21.0)	17 (15,20)*	16 (14,17.75)**	16.042
BNT^c^	26 (24,27)	24 (223,27)	23 (21.25,26)*	6.542
**Executive function**				
STT-A total time (sec) ^c^	40 (34,51)	44 (36,55)	52.5 (39.25,61.25)*	8.225
STT-B total time (sec) ^c^	113 (89,137)	116 (95,136)	127.5 (109.25,171.00)	
Stroop test A^c^	24 (24,24)	24 (24,24)	24 (24,24)	1.829
Stroop test B^c^	24 (23,24)	24 (23,24)	23 (20,24)	11.985
**Spatial Function**				
ST^a^	10.5 ± 2.61	9.7 ± 2.46	10.0 ± 2.16	1.206
JLO^a^	21.2 ± 5.04	20.7 ± 4.82	21.4 ± 4.32	0.186
**Attention function**				
DST sequence^c^	8 (7.5,8.5)	8 (6,8)	7 (5,8)*	8.837
DST reverse^c^	5 (4,6)	5 (4,5)	4.5 (4,5)	3.137

^*a*^ANOVA test, ^*b*^Chi-square test; ^*c*^Kruskal–Wallis H-test; *compared with HC group, p < 0.05; **compared with HC group, p < 0.001; *^†^*compared with SCD group, p < 0.05; *^‡^*compared with SCD group, p < 0.001. MMSE, Mini-Mental State Examination. ACE-III, Addenbrooke’s Cognitive Examination. MoCA-B, Montreal Cognitive Assessment-Basic. AVLT, Auditory Verbal Learning Test. BVMT, Brief Visuospatial Memory Test. AFT, Animal Verbal Fluency Test. BNT, Boston Naming Test. STT, Shape Trail Test. ST, Silhouettes Test. JLO, Judgment of Line Orientation. DST, Digit Span Test.

##### Comparison between the SCD group and the HC group

In the following neuropsychological tests, there were significant differences between the SCD group and the HC group: ACE-III [82 (77, 86) vs. 87 (82.5, 90.5), *p* = 0.002], VFT [17 (15, 20) vs. 19 (18.5, 21.0), *p* = 0.002] (Table 1).

##### Comparison between the aMCI group and the HC group

In the following neuropsychological tests, there were significant differences between the aMCI group and the HC group: MMSE [27 (26, 28) vs. 29 (28, 30), *p* = 0.002], ACE-III [75 (73, 81) vs. 87 (82.5, 90.5), *p* = 0.000], MoCA-B [23 (22, 25)

vs. 27 (26, 28), *p* = 0.000], AVLT immediate recall [12 (11, 14) vs. 18 (15.5, 20.5), *p* = 0.000], AVLT 4th recall [3 (2, 2.75) vs. 6 (5, 8), *p* = 0.000], AVLT 5th recall [2 (1, 3) vs. 6 (4.5, 7.5), *p* = 0.000], AVLT 6th recall [2 (2, 3) vs. 6 (5.0, 7.5), *p* = 0.000], AVLT recognition [18 (16, 18.75) vs. 22 (21, 23), *p* = 0.000], BVMT immediate recall [18 (14.25, 20.75) vs. 22 (16.5, 26), *p* = 0.036], BVMT-6th recall [4 (4, 4) vs. 5 (5, 6), *p* = 0.000], BVMT-recognition [12 (10, 12) vs. 12 (12, 12), *p* = 0.000], VFT [16 (14, 17.75) vs. 19 (18.5, 21.0), *p* = 0.000], BNT [23 (21.25, 26) vs. 26 (24, 27), *p* = 0.033], DST-sequence [7 (5, 8) vs. 8 (7.5, 8.5), *p* = 0.015] (Table 1).

##### Comparison between the aMCI group and the SCD group

In the following neuropsychological tests, there were significant differences between the aMCI group and the SCD group: ACE-III [75 (73, 81) vs. 82 (77, 86), *p* = 0.021], MoCA-B [23 (22, 25) vs. 27[26, 28), *p* = 0.007], AVLT immediate recall [12 (11, 14) vs. 19 (15, 21), *p* = 0.000], AVLT 4th recall [3 (2, 2.75) vs. 6 (5, 8), *p* = 0.000], AVLT 5th recall [2 (1, 3) vs. 6 (5, 7), *p* = 0.000], AVLT 6th recall [2 (2, 3) vs. 6 (4, 7), *p* = 0.000], AVLT recognition [18 (16, 18.75) vs. 22 (21, 24), *p* = 0.000], BVMT-6th recall [4 (4, 4) vs. 5 (5, 6), *p* = 0.000], BVMT-recognition [12 (10, 12) vs. 12 (12, 12), *p* = 0.001], STT-A total time [52.5 (39.25, 61.25) vs. 40 (34, 51), *p* = 0.012] (Table 1).

### rs-fMRI ReHo

#### ANOVA Analysis

**FIGURE 1:**
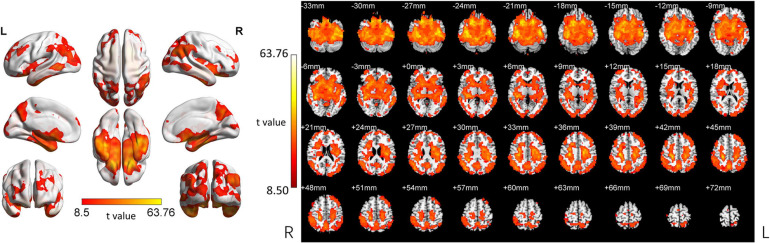
ANOVA revealed differences among the three groups. ReHo was significantly different among the three groups (Two-tailed ANOVA-test; FDR *p* < 0.001, *k* > 10 voxels). ANOVA, one way analysis of variance. The color bars indicate *t*-values, blue color represents negative values and red color represents positive values. L, Left; R, Right.

ReHo was significantly different between the three groups. The ANOVA showed that the differential brain regions were located in Temporal_Inf_L/R, Fusiform_L/R, Temporal_Sup_L/R, Insula_L/R, ParaHippocampal_L/R, Temporal_Pole_Sup_L/R, Hippocampus_L/R, Frontal_Inf_Orb_L/R, Temporal_Mid_L/R, Putamen_L/R, Caudate_L/R, Postcentral_L/R, Rolandic_ Oper_L, Precentral_L, Frontal_Sup_Orb_L/R, Rectus_R, Temporal_Pole_Mid_R, Precuneus_L/R, Occipital_Mid_L/R, Angular_L/R, Parietal_Inf_L/R, SupraMarginal_R, Parietal_ Sup_L/R, Occipital_Sup_L/R, and Cuneus_L/R (Two-tailed ANOVA-test; FDR *p* < 0.001, *k* > 10 voxels) (Figure 1 and Table 2).

**TABLE 2:** ANOVA revealed differences among three groups.

CLUSTER (AAL)	Volume (voxels)	CLUSTER (AAL)	Volume (voxels)
**Cluster 1**	Total: 22,347	**Cluster 2**	Total: 6,158
Peak (MNI): 39 -18 -24	Peak t: 63.7643	Peak (MNI): 3 -66 45	Peak t: 41.1763
Temporal_Inf_L	584	Precuneus_L	499
Temporal_Inf_R	495	Occipital_Mid_L	448
Fusiform_R	458	Precuneus_R	448
Fusiform_L	438	Angular_R	408
Temporal_Sup_L	392	Occipital_Mid_R	328
Insula_R	358	Temporal_Mid_R	315
Insula_L	340	Parietal_Inf_R	311
ParaHippocampal_R	325	Temporal_Mid_L	311
Cerebelum_4_5_L	315	Parietal_Inf_L	271
Cerebelum_6_R	299	SupraMarginal_R	244
ParaHippocampal_L	283	Parietal_Sup_L	235
Temporal_Pole_Sup_L	274	Angular_L	233
Hippocampus_R	268	Parietal_Sup_R	216
Hippocampus_L	267	Occipital_Sup_R	191
Frontal_Inf_Orb_L	251	Cuneus_R	168
Cerebelum_6_L	244	Cuneus_L	155
Frontal_Inf_Orb_R	242	Occipital_Sup_L	153
Temporal_Mid_L	228	Postcentral_R	110
Putamen_L	227		
Temporal_Sup_R	215		
Putamen_R	214		
Cerebelum_4_5_R	196		
Caudate_R	185		
Caudate_L	184		
Temporal_Pole_Sup_R	179		
Postcentral_L	172		
Rolandic_Oper_L	160		
Precentral_L	158		
Frontal_Sup_Orb_R	127		
Rectus_R	117		
Temporal_Pole_Mid_R	112		
Frontal_Sup_Orb_L	100		

*ANOVA, one way analysis of variance. AAL, anatomical automatic labeling atlas. MNI, Montreal Neurological Institute space. Only clusters of more than 100 voxels were reported.*

#### The SCD Group Compared With the HC Group

**FIGURE 2:**
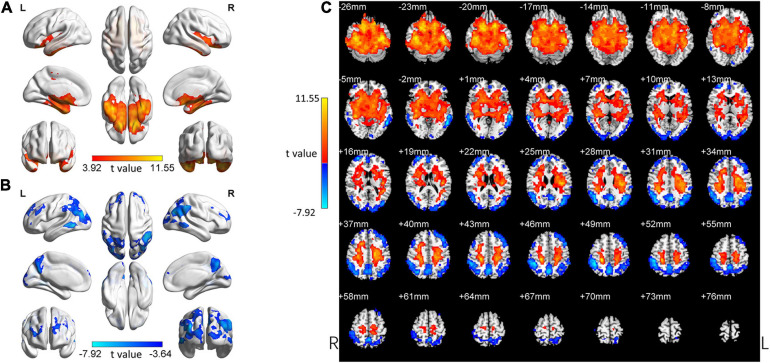
Changed ReHo in the SCD group compared with the HC group. **(A)** The brain regions with increased ReHo in the SCD group compared with the HC group. **(B)** The brain regions with decreased ReHo in the SCD group compared with the HC group. **(C)** Brain regions with increased and decreased ReHo in the SCD group compared with the HC group. Two-tailed *t*-test; FDR < 0.0005, *k* > 10 voxels. The color bars indicate *t*-values, blue color represents negative values and red color represents positive values. L, Left; R, Right.

In the following areas, the ReHo of the SCD group decreased compared to the HC group: Occipital_Mid_L/R, Precuneus_R, Angular_L/R, Parietal_Inf_L/R, Temporal_Mid_L/R, Parietal_ Sup_L/R, SupraMarginal_R, Occipital_Sup_L/R, Cuneus_L, Frontal_Mid_L/R, Frontal_Sup_L/R, and Frontal_Sup_Medial_L (Two-tailed, FDR *p* < 0.001, *k* > 10 voxels) (Figure 2 and Table 3). In the following areas, the ReHo of the SCD group increased compared to the HC group: Temporal_Inf_L/R, Fusiform_L/R, Temporal_Sup_L/R, Insula_L/R, Para- Hippocampal_L/R, Hippocampus_L/R, Frontal_Inf_Orb_L/R, Temporal_Pole_Sup_L/R, Putamen_L/R, Temporal_Mid_L, Caudate_L/R, Postcentral_L, Rolandic_Oper_L, Precentral_L, Frontal_Sup_Orb_R, Rectus_R, and Temporal_Pole_Mid_R (Two-tailed, FDR *p* < 0.0005, *k* > 10 voxels) (Figure 2 and Table 4).

**TABLE 3:** Brain regions with decreased ReHo in the SCD group and the aMCI group compared to the HC group.

SCD group < HC group		aMCI group < HC group	
Cluster (AAL)	Volume (voxels)	Cluster (AAL)	Volume (voxels)
**Cluster 1**	Total: 5122	**Cluster 1**	Total: 889
Peak (MNI): 45 -45 48	Peak t: -7.9188	Peak (MNI): 48 24 36	Peak t: -7.4097
Precuneus_L	446	Frontal_Mid_R	423
Occipital_Mid_L	412	Frontal_Sup_R	219
Precuneus_R	382	**Cluster 2**	total: 4706
Angular_R	348	Peak: 3 -63 45	Peak t: -9.9902
Parietal_Inf_L	328	Precuneus_L	429
Parietal_Inf_R	306	Angular_R	408
Temporal_Mid_R	270	Precuneus_R	393
Temporal_Mid_L	267	Parietal_Inf_R	282
Occipital_Mid_R	261	Temporal_Mid_L	277
Parietal_Sup_L	244	Occipital_Mid_L	257
Angular_L	223	SupraMarginal_R	250
SupraMarginal_R	178	Angular_L	224
Parietal_Sup_R	154	Temporal_Mid_R	211
Occipital_Sup_R	135	Occipital_Mid_R	187
Cuneus_L	124	Parietal_Inf_L	174
Occipital_Sup_L	110	Parietal_Sup_R	148
**Cluster 2**	total: 503	Parietal_Sup_L	142
Peak: 33 57 15	Peak t: -7.0202	Occipital_Sup_L	133
Frontal_Mid_R	321	Occipital_Sup_R	125
Frontal_Sup_R	106	Cuneus_R	121
**Cluster 3**	total: 1021	**Cluster 3**	total: 527
Peak: -42 30 39	Peak t: -6.6576	Peak (MNI): -42 33 36	Peak t: -6.5666
Frontal_Mid_L	352	Frontal_Mid_L	273
Frontal_Sup_L	172	Frontal_Sup_L	101
Frontal_Sup_Medial_L	144		

*AAL, anatomical automatic labeling atlas. MNI, Montreal Neurological Institute space. Only clusters of more than 100 voxels were reported.*

**TABLE 4:** Brain regions with increased ReHo in the SCD group and the aMCI group compared to the HC group.

SCD group > HC group		aMCI group > HC group	
Cluster (AAL)	Volume (voxels)	Cluster (AAL)	Volume (voxels)
**Cluster 1**	Total: 21,873	**Cluster 1**	Total: 22,001
Peak (MNI): -42 -18 -24	Peak t: 11.5543	Peak (MNI): 36 -33 -24	Peak t: 11.7286
Temporal_Inf_L	558	Temporal_Inf_L	590
Fusiform_R	452	Temporal_Inf_R	486
Temporal_Inf_R	438	Fusiform_R	442
Fusiform_L	432	Fusiform_L	413
Temporal_Sup_L	379	Temporal_Sup_L	359
Insula_R	344	Insula_R	319
Insula_L	318	ParaHippocampal_R	310
Cerebelum_4_5_L	311	Cerebelum_4_5_L	298
ParaHippocampal_R	308	Cerebelum_6_R	281
Cerebelum_6_R	292	ParaHippocampal_L	281
ParaHippocampal_L	280	Temporal_Pole_Sup_L	273
Hippocampus_L	266	Insula_L	272
Hippocampus_R	256	Hippocampus_L	268
Frontal_Inf_Orb_R	252	Hippocampus_R	263
Temporal_Pole_Sup_L	251	Cerebelum_6_L	227
Frontal_Inf_Orb_L	240	Frontal_Inf_Orb_L	226
Cerebelum_4_5_R	229	Frontal_Inf_Orb_R	225
Cerebelum_6_L	223	Putamen_L	217
Putamen_R	221	Temporal_Mid_L	214
Putamen_L	221	Putamen_R	201
Temporal_Mid_L	214	Temporal_Sup_R	200
Caudate_L	208	Caudate_R	189
Caudate_R	191	Cerebelum_4_5_R	184
Temporal_Sup_R	188	Caudate_L	173
Postcentral_L	159	Temporal_Pole_Sup_R	162
Temporal_Pole_Sup_R	158	Postcentral_L	126
Rolandic_Oper_L	158	Frontal_Sup_Orb_R	126
Precentral_L	152	Precentral_L	125
Frontal_Sup_Orb_R	122	Rectus_R	120
Rectus_R	109	Rolandic_Oper_L	120
Vermis_4_5	102	Cerebelum_8_L	119
Temporal_Pole_Mid_R	100	Cerebelum_9_R	117
		Frontal_Sup_Orb_L	104
		Temporal_Pole_Mid_R	101

*AAL, anatomical automatic labeling atlas. MNI, Montreal Neurological Institute space. Only clusters of more than 100 voxels were reported.*

#### The aMCI Group Compared With the HC Group

Comparing the aMCI group with the HC, the brain area with ReHo changes was very similar to the SCD group (Tables 3, 4).

**FIGURE 3:**
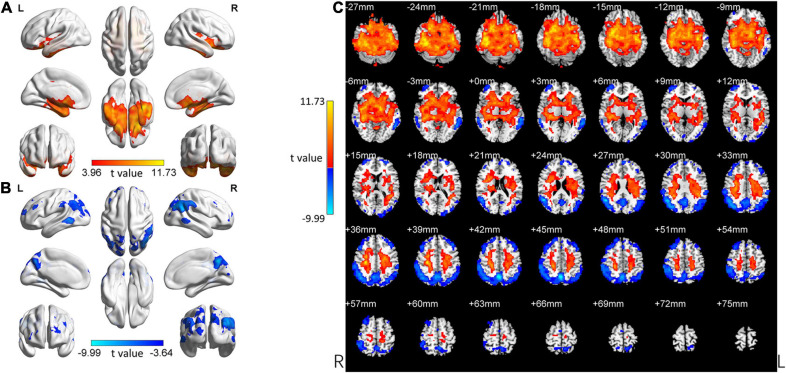
Changed ReHo in the aMCI group compared with the HC group. **(A)** The brain regions with increased ReHo in the aMCI group compared with the HC group. **(B)** The brain regions with decreased ReHo in the aMCI group compared with the HC group. **(C)** Brain regions with increased and decreased ReHo in the aMCI group compared with the HC group. Two-tailed *t*-test; FDR < 0.0005, *k* > 10 voxels. The color bars indicate *t*-values, blue color represents negative values and red color represents positive values. L, Left; R, Right.

In the following areas, the ReHo of the aMCI group decreased compared to the HC group: Frontal_Mid_L/R, Frontal_Sup_L/R, Precuneus_L, Angular_L/R, Precuneus_R, Parietal_Inf_L/R, Temporal_Mid_L/R, Occipital_Mid_L, SupraMarginal_R, Occipital_Mid_R, Parietal_Sup_L/R, Occipital_Sup_L/R, and Cuneus_R (Two-tailed, FDR *p* < 0.001, *k* > 10 voxels) (Figure 3 and Table 3). Bonferroni’s correction was used for *Post-hoc* analyses. In the following areas, the ReHo of the aMCI group increased compared to the HC group: Temporal_Inf_L/R, Fusiform_L/R, Temporal_Sup_L/R, Insula_L/R, ParaHippocampal_L/R, Temporal_Pole_Sup_L/R, Hippocampus_L/R, Frontal_Inf_Orb_L/R, Putamen_L/R, Temporal_Mid_L, Caudate_L/R, Postcentral_L, Frontal_Sup_ Orb_L/R, Precentral_L, Rectus_R, Rolandic_Oper_L, and Temporal_Pole_Mid_R (Two-tailed, FDR *p* < 0.0005, *k* > 10 voxels) (Figure 3 and Table 4).

#### Similarity Between the aMCI Group and the SCD Group

Our study focused on comparing the SCD group and the aMCI group with the HC group to explore the change patterns of these two groups (SCD and aMCI group). We visually observed the change patterns in the SCD group and the aMCI group and found these two groups were very similar (Figures 2, 3).

### Correlation Analysis

#### Define ROI

In the SCD group, the significant cluster with increased ReHo (Table 3, SCD group > HC group, cluster 1, peak coordinate: -42 -18 -24) was defined as increased ReHo ROI (IRR). The most significant cluster with decreased ReHo (Table 4, SCD group < HC group, cluster 1, Peak coordinate: 45 -45 48) was defined as decreased ReHo ROI (DRR). The ReHo values of these two ROIs were extracted and analyzed with spearmen rank correlation.

#### Correlation Between ReHo With Neuropsychological Performances

**FIGURE 4:**
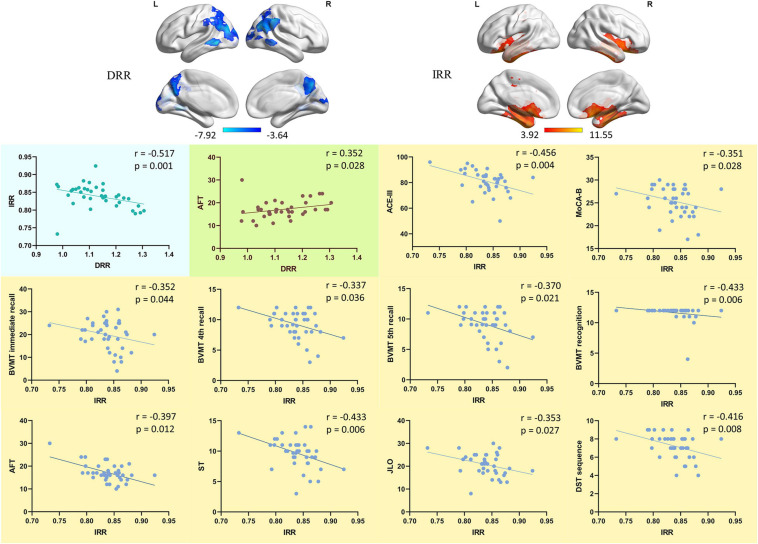
The correlation of DRR/IRR and neuropsychological performance. The correlation between the ReHo value of DRR/IRR and the neuropsychological tests performance. DRR, decreased ReHo region. IRR, increased ReHo region. ACE-III, Addenbrooke’s Cognitive Examination. MoCA-B, Montreal Cognitive Assessment-Basic. BVMT, Brief Visuospatial Memory Test. AFT, Animal Verbal Fluency Test. ST, Silhouettes Test. JLO, Judgment of Line Orientation. DST, Digit Span Test.

The ReHo value of DRR and IRR was significantly negatively correlated (*r* = −0.517, *p* = 0.001) (Spearman rank correlation, two-tailed) (Figure 4). The ReHo value of DRR was significantly positively correlated with AFT (*r* = 0.352, *p* = 0.028) (Spearman rank correlation, two-tailed) (Figure 4). The ReHo value of IRR was significantly negatively correlated with ACE-III (*r* = −0.456, *p* = 0.004), MoCA-B (*r* = −0.351, *p* = 0.028), BVMT immediate recall (*r* = −0.352, *p* = 0.044), BVMT 4th recall (*r* = −0.337, *p* = 0.036), BVMT 5th recall (*r* = −0.370, *p* = 0.021), BVMT recognition (*r* = −0.433, *p* = 0.006), AFT corrections (*r* = −0.397, *p* = 0.012), ST (*r* = −0.433, *p* = 0.006), JLO (*r* = −0.353, *p* = 0.027), DST sequence (*r* = −0.416, *p* = 0.008) (Spearman rank correlation, two-tailed) (Figure 4).

## Discussion

Prior studies have noted the importance of SCD. Although it is generally believed that neuropathological changes have occurred at this stage, it is still difficult to detect such changes non-invasively. The first question in this study sought to determine is whether it is possible to find changes in neural activity homogeneity in the brains of SCD patients and the characteristics of these changes in different brain regions. If these changes did exist, the second question this study aimed to address was whether they were associated with cognitive decline. The third question we wanted to discuss was the similarity and significance of these changes in patients with SCD and aMCI. Regarding the first question, we found that in SCD patients, there were increased and decreased ReHo in several brain regions, suggesting that these regions had changed neurological activity homogeneity. Besides, there was a correlation between the increased and decreased areas of ReHo. On the second question, we found correlation between changed ReHo and neuropsychological performance, suggesting that the homogeneity of neural activity may be related to cognitive ability. At last, it was worth noting that the areas with changed ReHo in the SCD group were quite similar to the aMCI patients. These results suggest that regional neural activity homogeneity may be a potential biomarker for identifying SCD and measuring the disease severity.

In the ANOVA analysis of rs-fMRI, ReHo changes in a wide range of brain areas were shown among the three groups. Further comparisons between groups showed that compared with the HC group, both the SCD group and aMCI group showed similar and regular changes in brain areas. In the SCD group, the ReHo decreased in part of the parietal lobe, frontal lobe, occipital lobe, and temporal lobe. The correlation analysis showed that the ReHo of DRR was positively correlated with AFT scores, suggesting that the decrease of neural activity homogeneity in these areas may be related to the impairment of language fluency. Most of these involved areas have been confirmed to be related to cognitive function in previous studies. The parietal lobe is a critical node for integrating cognitive activities; although recruitment is distributed in multiple brain regions in every cognitive activity, the parietal lobe is a converging area. A study (Bzdok et al., 2016) found that the function of rostro-ventral and caudo-ventral regions in the parietal lobe was significantly associated with social-cognitive and language processing. In the frontal lobe and cognition related field, a study on MCI (Garcia-Alvarez et al., 2019) found that damage to the cortex and functional circuits of the frontal lobe is associated with a decline in working memory and executive function, as well as with the loss of daily function. A study using near-infrared spectroscopy (Chaudhary et al., 2011) revealed that the oxy-hemoglobin increased during the verbal fluency task while the deoxy-hemoglobin decreased in the frontal cortex. Although the occipital lobe is mainly involved in visual function, recent studies have suggested that the occipital lobe is also associated with cognitive decline in MCI and AD patients. A multimodal imaging study in healthy group, MCI group, and AD group showed that the number of connections between brain regions gradually decreased, especially in the occipital-parietal lobe (Li et al., 2018). Another study focused on cholinergic impairment in MCI patients found that Acetylcholinesterase activity was mainly reduced in the lateral temporal cortex and the occipital lobe (Richter et al., 2019). These studies suggest that the occipital lobe and cognitive impairment relationship are probably closely related to the parietal and temporal lobes. Our results matched those observed in earlier studies. It is worth noting that the ReHo value of DRR seems to be only correlated with verbal fluency, and no obvious correlation with other cognitive domains has been found. This suggests that the decrease of neural homogeneity in these areas may not independently reflect the degree of cognitive impairment. In previous studies on SCD, it has been found that these areas, such as the parietal and frontal lobes, play a role in the cognitive decline of SCD mainly through abnormal connections with other parts of the brain or networks (Viviano et al., 2019; Wang et al., 2019).

It is somewhat surprising that our study found a much larger area with significantly increased ReHo in the SCD group. These areas are centered on the temporal lobe and extend to the adjacent part of the occipital lobe, parietal lobe, and subcortical structures. The temporal lobe’s structural changes, especially in the medial temporal lobe and the hippocampus, have been identified as typical MRI markers of AD (Scheltens et al., 2016; Lane et al., 2018). In a study using FDG-PET (Pagani et al., 2017), researchers established a cohort of MCI due to AD through longitudinal follow-up. They found that in MCI patients who eventually converted to AD, FDG uptake was lower in temporal and parietal cortices; in MCI patients who did not convert to AD, the FDG intake in these areas did not change. This study further confirmed that the temporal lobe and parietal lobe play an essential role in predicting MCI patients’ development to AD. Some PET studies using tracers for tau protein found uptake in orbitofrontal, parietal, hippocampal, and temporal cortices in humans with AD (Fodero-Tavoletti et al., 2011; Villemagne et al., 2014). An amyloid PET study found a high binding affinity for Aβ in the frontal, temporal, and posterior cingulated cortices in AD patients (Maya et al., 2016). These PET studies using special tracers provide pathological evidence for multiple brain areas dominated by the temporal lobes. In addition, some studies (Hilal et al., 2015; Koshiyama et al., 2018) have revealed the role of specific subcortical nuclei in cognitive and social function, and these observations were similar to our results.

Due to the resolution and sensitivity characteristics, no consistent conclusion of studies using PET in the preclinical stage of AD has been reached so far. Structural damage often occurs at a later stage; therefore, it is also difficult to locate specific damaged brain regions of SCD patients through structural MRI. Rs-fMRI has a high spatial resolution and sensitivity to changes in neural activity and function. Moreover, it does not need to perform tasks to stimulate neural activities, which makes rs-fMRI has good stability and repeatability. We had predicted that compensatory phenomena in the brain of patients with SCD could be detected by rs-fMRI, but the scope of compensation found in the study is beyond our expectations. We cautiously speculate that this compensation may be one reason why SCD patients can retain roughly normal cognitive abilities. The results of the subsequent correlation analysis confirmed this speculation to a certain extent. We analyzed the correlation between the ReHo value of IRR (the ROI representing the increased ReHo area) and neuropsychological test performance, found that the ReHo of IRR was negatively correlated with multiple neuropsychological tests performance. These neuropsychological tests include ACE-III and MoCA-B reflecting general cognitive function; BMVT reflecting memory function; AFT and ST reflecting language function; JLO reflecting spatial function; and DST reflecting attention function. These different cognitive domains’ performance decreased with the increase of the Reho value of IRR, indirectly indicating that the increase in local neural activity homogeneity in these areas reflects brain damage aggravation. ReHo has shown the relation with brain function compensation in different studies (Chen et al., 2016; Guo et al., 2016), suggesting that we should pay attention to the role of ReHo in the research of relatively mild diseases that may have functional compensation.

Compared with the HC group, the distribution of brain regions with changed ReHo in the SCD and aMCI groups was similar. We think there might be multiple possible reasons. The first reason is that SCD is the latest stage of preclinical AD, and its outcome is likely to be aMCI. The two stages are closely linked in the course of the disease, so there is likely to be a significant similarity in neurological damage manifestations. The second reason is that the primary method used to distinguish these two stages is neuropsychological testing. Commonly used neuropsychological tests may not have sufficient sensitivity and specificity in the early stage of the disease; therefore, there may be some overlap in the diagnosis of these two stages to a certain extent (Jessen et al., 2020). The third reason is that our research participants were recruited through advertising and volunteered for a full set of complex and lengthy tests. These participants who actively participated in the study may have more concerns about memory complaints, which may cause the participants in the SCD group to be more severe than the cohort obtained by community screening. This reason may aggravate the problem of diagnosis overlap caused by the second reason. Therefore, the results of our research need to be interpreted and promoted cautiously. Our study found that although certain brain areas of MCI patients also showed improved local neurological uniformity similar to those of SCD patients, their neuropsychological test performance was significantly lower than that of SCD patients. This suggests that this kind of compensation is limited, and there is a ceiling effect. When the disease reaches a certain level, this compensation will not be able to maintain the patient’s relatively normal cognitive function.

## Conclusion

We used rs-fMRI to study the regional neural activity homogeneity of SCD patients’ brains and found significant changes. Part of the parietal, frontal, and occipital lobes showed decreased neural homogeneity and positively correlated with some cognitive domains’ decline. The temporal lobe, part of the parietal lobe and frontal lobe, showed an increase in neural homogeneity. The ReHo value of the area with increased neural homogeneity is negatively correlated with multiple neuropsychological tests’ performance, suggesting that the increased regional neural activity homogeneity in SCD patients may be a compensatory manifestation of neural damage. Simultaneously, the neurological homogeneity of SCD patients is similar to that of aMCI patients, which confirms that patients in these two stages have similar neuropathy. However, the aMCI group’s cognitive function was significantly worse than that of the SCD group, suggesting that this compensation is limited. In summary, regional neural activity homogeneity may be a potential biomarker for identifying SCD and measuring the disease severity.

## Limitation

However, there are some limitations in this study, which can restrict the generalizability of our results. First, the sample size was not large enough, and AD patients were not included in the study. Second, although a complete set of neuropsychological tests was used, there might be some overlap between SCD and aMCI groups. Third, we did not perform the examination of pathological biomarkers. Considering the limitations mentioned above, the results of this study should be interpreted with caution. In follow-up research, We should expand the sample size, explore a more sensitive neuropsychological test diagnostic approach, and examine pathological biomarkers.

## Data Availability Statement

The raw data supporting the conclusions of this article will be made available by the authors, without undue reservation.

## Ethics Statement

The studies involving human participants were reviewed and approved by the Ethics Committee of Shanghai Jiao Tong University Affiliated Sixth People’s Hospital. The patients/participants provided their written informed consent to participate in this study.

## Author Contributions

ZZ: data analysis, data curation, revision. LC: methodology, data analysis, investigation, data curation, writing—original draft, visualization. YH: investigation, writing—review, editing. YC: review, commentary. YL: resources, project administration. QG: resources, supervision, project administration, funding acquisition, review, and revision. All authors contributed to manuscript revision, read, and approved the submitted version.

## Conflict of Interest

The authors declare that the research was conducted in the absence of any commercial or financial relationships that could be construed as a potential conflict of interest.

## Footnotes

1 http://www.fil.ion.ucl.ac.uk/spm/software/spm12/

2 http://restfmri.net/forum/restplus

3 http://www.nitrc.org/projects/bnv/

## Abbreviations

SCD: subjective cognitive decline
AMCI: amnestic mild cognitive impairment
Rs-fMRI: resting-state functional magnetic resonance imaging
ReHo: regional homogeneity
AD: Alzheimer’s disease
MMSE: Mini-Mental State Examination
ACE-III: Addenbrooke’s Cognitive Examination
MoCA-B: Montreal Cognitive Assessment-Basic
AVLT: Auditory Verbal Learning Test
BVMT: Brief Visuospatial Memory Test
AFT: Animal Verbal Fluency Test
BNT: Boston Naming Test
ST: Silhouettes Test
STT: Shape Trail Test
JLO: Judgment of Line Orientation
DST: Digit Span Test
MNI: Montreal Neurological Institute
ROI: Region of interest
DRR: decreased ReHo region
IRR: increased ReHo region
AAL: anatomical automatic labeling atlas.
